# Clinical and Molecular Characteristics of Gonadotroph Pituitary Tumors According to the WHO Classification

**DOI:** 10.1007/s12022-023-09794-w

**Published:** 2023-12-14

**Authors:** Francesca Carbonara, Tiziana Feola, Francesca Gianno, Michela Anna Polidoro, Rosa Maria Di Crescenzo, Antonietta Arcella, Michelangelo De Angelis, Roberta Morace, Dario de Alcubierre, Vincenzo Esposito, Felice Giangaspero, Marie-Lise Jaffrain-Rea

**Affiliations:** 1 Department of Biotechnological and Applied Clinical Sciences, University of L’Aquila, L’Aquila, Italy; 2 Neuromed IRCCS, Pozzilli, Italy; 3 Department of Experimental Medicine, La Sapienza University of Rome (RM), Rome, Italy; 4 Department of Radiological, Oncological and Pathological Sciences, La Sapienza University of Rome (RM), Rome, Italy; 5 Hepatobiliary Immunopathology, Humanitas Clinical and Research Center IRCCS, Rozzano, Italy; 6 Department of Advanced Biomedical Sciences, Pathology Section, University of Naples Federico II, Naples, Italy; 7 Department of Neurology and Psychiatry, La Sapienza University of Rome (RM), Rome, Italy

**Keywords:** Pituitary neuroendocrine tumors (PitNETs), Gonadotroph tumors, SF1, Dopamine receptor 2 (D2R), Cell cycle, Aryl hydrocarbon receptor interacting protein (AIP)

## Abstract

Since 2017, hormone-negative pituitary neuroendocrine tumors expressing the steroidogenic factor SF1 have been recognized as gonadotroph tumors (GnPT) but have been poorly studied. To further characterize their bio-clinical spectrum, 54 GnPT defined by immunostaining for FSH and/or LH (group 1, *n* = 41) or SF1 only (group 2, *n* = 13) were compared and studied for *SF1*, *βFSH*, *βLH*, *CCNA2*, *CCNB1*, *CCND1*, *caspase 3*, *D2R*, and *AIP* gene expression by qRT-PCR. Immunohistochemistry for AIP and/or D2R was performed in representative cases. Overall, patients were significantly younger in group 1 (*P* = 0.040 vs group 2), with a similar trend excluding recurrent cases (*P* = 0.078), and no significant difference in gender, tumor size, invasion or Ki67. *SF1* expression was similar in both groups but negatively correlated with the patient’s age (*P* = 0.013) and positively correlated with *βLH* (*P* < 0.001) expression. *Beta-FSH* and *AIP* were significantly higher in group 1 (*P* = 0.042 and *P* = 0.024, respectively). Ki67 was unrelated to gonadotroph markers but positively correlated with *CCNB1* (*P* = 0.001) and negatively correlated with *CCND1* (*P* = 0.008). *D2R* and *AIP* were strongly correlated with each other (*P* < 0.001), and both positively correlated with *SF1*, *βFSH*, *βLH*, *and CCND1*. AIP immunopositivity was frequently observed in both groups, with a similar median score, and unrelated to Ki67. D2R immunostaining was best detected with a polyclonal antibody and mostly cytoplasmic. This study indicates that hormone-negative GnPT tend to occur in older patients but do not significantly differ from other GnPT in terms of invasion or proliferation. It also points out the current limits of D2R immunostaining in such tumors.

**Supplementary Information:**

The online version contains supplementary material available at 10.1007/s12022-023-09794-w.

## Introduction

A large subset of pituitary neuroendocrine tumors (PitNETs) derives from gonadotroph cells. Most gonadotroph PitNETs (GnPT) do not secrete sufficient amounts of biologically active hormones to determine clinical features of hypersecretion and present as non-functioning pituitary tumors (NFPT) revealed by mass effects and/or endocrine dysfunction, including hypogonadism [1, 2]. Indeed, hypergonadism is rare, and the ovarian hyperstimulation syndrome was estimated to occur in about 3% of pre-menopausal women with a NFPT and 8% in those displaying immunoreactivity for gonadotropins [2]. Thus, most cases are diagnosed after surgery, based on immunohistochemical staining for gonadotropins and/or, since 2017, the steroidogenic factor 1 (SF1) [3, 4]. SF1 (also known as NR5A1), a transcription factor involved in the differentiation of gonadotroph cells, is currently considered the most sensitive and specific diagnostic marker of GnPT [5] and is especially useful in hormone-negative PitNETs or in the presence of equivocal staining for gonadotropins [6]. In a very large surgical series of PitNETs (*n* > 1000), up to 2/3 of hormone-negative PitNETs turned out to express SF1 only, thereby increasing the proportion of GnPT from 58 to 73% [7]. Overall, the molecular basis of GnPT has been less extensively studied than in other pituitary tumor subtypes [8], and the current knowledge would benefit from some revisions based on the current pathological definition of GnPT. To the best of our knowledge, potential bio-clinical differences between FSH/LH immuno-positive and pure SF1-expressing (pSF1) GnPT have not been specifically addressed.

The first therapeutic option in GnPT is surgery, and in the presence of post-operative tumor regrowth, re-operation and/or radiotherapy are generally recommended [1]. Indeed, no drug is currently approved for their medical treatment, although a subset of clinically NFPT may benefit from dopamine-agonists (DA), in particular cabergoline (CAB), with a moderate tumor shrinkage and/or stabilization reported in a majority of patients [9]. DA have also been used with some success in functional GnPT [10]. For such reasons, DA may be proposed as an alternative to radiotherapy in selected NF/GnPT after surgery [9]. Tumor shrinkage in NFPT has been variably associated with the expression of the dopamine receptor type 2 (D2R) [11–13]. However, factors influencing D2R expression in GnPT are poorly known.

In previous studies, we and other authors reported that the aryl hydrocarbon receptor interacting protein (AIP), which was identified in 2006 as a tumor suppressor gene in GH- and/or PRL-secreting PitNETs [14] and currently represents the main predisposing gene for the development of such tumors [15, 16], could be paradoxically overexpressed in NF/GnPT [17, 18]. Indeed, the pituitary expression of AIP was found to be normally restricted to somatotrophs and lactotrophs [17, 18], suggesting the presence of abnormal mechanisms of AIP regulation in GnPT. GnPT are also less frequently reported in familial isolated pituitary adenomas (FIPA) or in the presence of germline *AIP* mutations [15]. AIP overexpression may be essentially limited to a subset of GnPT with a high Ki67 [19]. However, this has not been confirmed yet, and the biological significance of AIP expression in GnPT has not been further explored.

In this study, we aimed to provide new insight in some molecular characteristics of GnPT according to the expression of FSH/LH and/or SF1 only. We focused our attention on the relationship between *SF1, βLH*, and *βFSH* gene expression and representative markers of cell cycle, as well as *D2R* and *AIP*. We also attempted to further characterize pSF1 tumors as compared to other GnPT from a translational point of view.

## Material and Methods

### Patients and Tumors

Surgical biopsies of 54 GnPT operated on at the Neuromed Institute (Pozzilli, IS, Italy) were studied. All patients (37 M, 17 F, median age 59 years, range 36–83) were operated on for medical reasons, most of them by a transsphenoidal route (*n* = 52). For each patient, pre-operative data were collected, including hormone assays (PRL, FSH, LH, testosterone in males, estradiol in females) and magnetic resonance imaging (MRI). All were macrotumors (maximal diameter > 1 cm), including 12 giant tumors (≥ 4 cm). Invasiveness was defined according to pre-operative MRI and intra-operative findings. Immunohistochemistry was carried out on 4-μm thickness sections, using an automatic immunostaining Benchmark ultra XT (Ventana), and an Ultra View DAB Detection Kit (Roche Diagnostic) for antibody signal detection. Immunohistochemical staining was obtained for all pituitary hormones, Ki67, and SF1 as appropriate [6]. The pathological diagnosis of GnPT was based on the WHO classification [3, 4] and tumors were divided into 2 groups according to their immunohistochemical profile: group 1 (FSH/LH), group 2 (pSF1). The following antibodies and conditions were used for diagnostic purposes: βFSH (prediluted, Roche), βLH (prediluted, Roche), SF1 (Ab217317 dil 1:500), and Ki67 (MIB1 antibody, Diagnostic Brokers Associated, Milan, Italy). The Ki67 labeling index of cell proliferation (Ki67 LI) was calculated on 500 to 1000 cells after image acquisition on a camera and manual count, considering hotspot areas where present [6]. A cut-off of 3% was used to define “high Ki67” tumors (≥3%) or “low Ki67” tumors (<3%). Surgical samples from 3 normal post-mortem pituitaries (NP) were used for all gene expression studies, whereas a small number of functional lactotroph tumors (clinically defined before surgery and confirmed by unequivocal and exclusive PRL immunostaining) were used as controls for *D2R* gene expression (*n* = 13) and/or protein expression by immunohistochemistry (IHC) (*n* = 9). The study was performed according to the guidelines of the Declaration of Helsinki and approved by the Internal Review Board of the Neuromed Institute (Pozzilli, Italy). Written informed consent was obtained from the patients, except for a minority of archive RNA or paraffin-embedded material from patients lost to follow-up.

### Gene Expression Analysis

Surgical biopsies were collected in RNAlater stabilization solution (Ambion^®^, Life Technologies, Monza, Italy) at room temperature and subsequently frozen at −80 °C until use. Total RNA was extracted by TRIzol™ Reagent (Ambion^®^, Life Technologies, Monza, Italy). After DNAse treatment (New England Biolabs), 500 ng of RNA-treated solution was reverse transcribed with Wonder RT (Euroclone, Pero, Italy) according to the manufacturer’s instructions. Preliminary RT-PCR amplification of GAPD(H) was performed to ensure cDNA quality, including PCR on total DNAse-treated RNA to exclude the presence of genomic DNA. RT-PCR for Pit-1 and Tpit was performed in order to exclude sample contamination by normal pituitary as previously described [20]. Gene expression was studied by semi-quantitative Real-Time RT-PCR using the Taqman methodology on an Applied Biosystems 7500 Fast Real-Time PCR and ready-to-use gene expression assays (Applied Biosystems, Life Technologies, Monza, Italy) for genes encoding SF1/NR5A1 (Hs00610436_m1), βFSH (Hs00174919_m1), βLH (Hs00751207_m1), cyclin D1 (*CCND1*, Hs00765553_m1), cyclin A2 (*CCNA2,* Hs00996788_m1), cyclin B1 (*CCNB1*, Hs01030099_m1), caspase 3 (Hs00234387_m1), AIP (Hs00610222_m19), D2R (Hs00241436_m1), and β-actin (Hs_99999903) as a house-keeping gene. The Taqman probe for D2R recognized both the short and long isoforms of D2R transcripts. All experiments were run at least in duplicate.

### Immunohistochemistry (IHC)

IHC was performed on paraffin-embedded sections as described hitherto. Two commercial antibodies were tested for D2R expression: a monoclonal antibody (mab9266–100, R&D system, distributed by Aurogene, Italy, referred to as D2R-mAb, dilution 1:100) and a polyclonal antibody (AB5084P, Sigma-Aldrich, distributed by Sial, Italy, referred to as D2R-pAb, dilution 1:500), the latter being previously used in this indication [12, 13]. Brain sections from epilepsy surgery (temporal lobe) were primarily used as positive controls to set experimental conditions for both antibodies, and these were subsequently tested on normal pituitary fragments contaminating pituitary tumor samples. Endothelial cell immunostaining was also used as an internal control for D2R protein expression (D2R-pAb). A monoclonal anti-AIP (clone 35–2, NOVUS Biologicals LLC, Littleton, CO, USA) was used as previously described, using a similar semiquantitative-based score and the intensity and pattern of AIP immunostaining (score 0–6) [21]. AIP immunopositivity was defined by a score > 2 [19, 21] and high AIP-IHC by an AIP score > 4. A qualitative evaluation was provided for D2R immunostaining*.*

### Statistical Analysis

Statistical analysis was performed using GraphPad Prism v10.0.2. Continuous data were expressed in median (range) and analyzed by non-parametric tests: Mann–Whitney test for two groups-analysis, Kruskal–Wallis for multiple comparisons, and Spearman test for correlation studies. One-tailed or two-tailed analyses were performed for two group comparisons, as appropriated. Categorical values were compared by the Chi-square test. *P* < 0.05 was considered significant.

## Results

### General Results

**Fig. 1:**
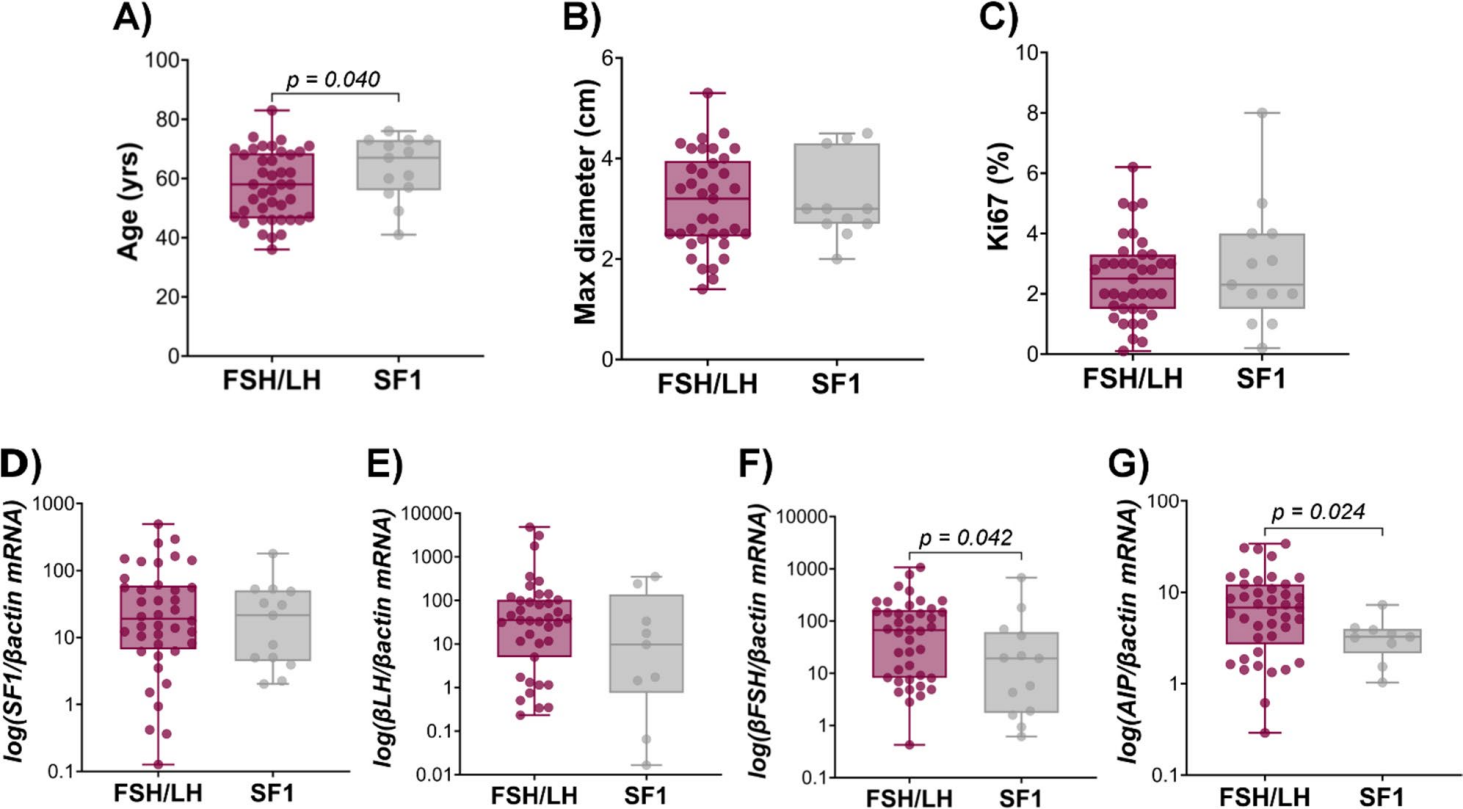
Bio-clinical characteristics of GnPT according to the presence or absence of gonadotropin immunostaining. Patients with FSH and/or LH immunostaining GnPT were significantly younger than those operated for pSF1 tumors (**A**), with no difference in tumor size (**B**) or Ki67 labeling index (%) (**C**) between the 2 groups. A significantly higher expression of *βFSH* and *AIP* was observed in the first group (**D**–**G**)

The main clinical, pathological, and molecular characteristics of patients and tumors are summarized in Table 1 and illustrated in Fig. 1A–C. A male predominance was observed in both groups, and at the time of surgery, patients were significantly younger in group 1 (median age 58 vs 67 years in group 2, *P* = 0.040). A similar trend was found excluding recurrent tumors, which could impact on patients’ age (median age 54 years in group 1 vs 61 years in group 2, *P* = 0.078). No significant differences were found between the two groups in terms of tumor size, invasiveness, and proliferation index. Details about data obtained in recurrent tumors are provided in Supplemental Table 1.

**Table 1:** Bio-clinical and molecular characteristics of GnPT according to the presence or the absence of gonadotropin immunostaining

	**FSH/LH**	**SF1**	***P***
**I. Clinicopathological data**			
Number of patients	41	13	
Gender	30 M; 11 F	7 M; 6 F	ns
Age (years)	58 (36–83)	67 (41–76)	0.040
Tumor size [cm]	3.20 [1.40–5.30]	3.00 [2.00–4.50]	ns
Giant (%)	9/40 (22.5%)	3/13 (23.1%)	ns
Invasive tumors	25/40 (62.5%)	7/13 (53.8%)	ns
Recurrent	4/41 (9.8%)	3/13 (23.1%)	ns
Ki67 (%)	2.50 (0.10–6.20)	2.30 (0.20–8.0)	ns
**II. Gene expression**			
*SF1/βactin* mRNA	19.1 [0.13–492.0]	21.4 [2.02–179]	ns
*βFSH/βactin* mRNA	66.9 [0.43–1067]	19.2 [0.61–680]	0.042
*βLH/βactin* mRNA	35.0 [0.23–4820]	9.74 [0.02–351]	ns
*CCND1/βactin* mRNA	16.0 [1.18–98.1]	18.2 [1.94–115]	ns
*CCNA2/βactin* mRNA	0.07 [0.01–1.27]	0.08 [0.01–1.81]	ns
*CCNB1/βactin* mRNA	0.57 [0.05–2.75]	0.70 [0.11–1.78]	ns
*Caspase 3/βactin* mRNA	0.32 [0.01–1.36]	0.15 [0.01–1.98]	ns
*AIP/βactin* mRNA	6.80 [0.29–33.9]	3.25 [1.03–7.27]	0.024
*D2R/βactin* mRNA	7.71 [0.08–213]	3.85 [0.44–49.2]	ns
**III. Immunohistochemistry**			
D2R mAb^a^	1/11 (9.0%)	1/4 (25.0%)	ns
D2R pAb^a^	9/13 (69.2%)	1/2 (50.0%)	ns
AIP^a^	25/34 (73.5%)	4/6 (66.7%)	ns
AIP score	3 [1–6]	3 [1–4]	ns

^a^Positive immunostaining

### Relationship Between Tumor SF1mRNA and the Clinical/Molecular Characteristics of GnPT

From a clinical point of view, an inverse and significant relationship was observed between *SF1* transcripts and patients’ age (*r* = −0.339, *P* = 0.013), and a similar relationship was found excluding recurrent cases (*r* = −0.382, *P* = 0.013) (Table 2). Accordingly, SF1 transcripts were significantly lower in tumors operated from patients aged > 65 years at the time of surgery as compared to younger patients (*P* = 0.008) (Supplemental Fig. 1). In contrast, no significant difference was found in *SF1* expression according to gender, tumor size, or invasiveness (data not shown).

**Table 2:** Gene expression data obtained in 54 GnPT: correlation matrix

		SF1 mRNA	βFSH mRNA	βLH mRNA	CCND1 mRNA	CCNA2 mRNA	CCNB1 mRNA	Caspase 3 mRNA	AIP mRNA	D2R mRNA	Ki67	Age
SF1 mRNA	Spearman’s rho	–										
	*p* value	–										
βFSH mRNA	Spearman’s rho	0.186	–									
	*p* value	0.181	–									
βLH mRNA	Spearman’s rho	0.506	0.144	–								
	*p* value	**< .001**	0.328	–								
CCND1 mRNA	Spearman’s rho	0.267	0.222	0.346	–							
	*p* value	0.067	0.125	**0.024**	–							
CCNA2 mRNA	Spearman’s rho	0.209	−0.174	0.148	0.064	–						
	*p* value	0.172	0.253	0.342	0.678	–						
CCNB1 mRNA	Spearman’s rho	−0.008	−0.067	0.094	−0.137	0.342	–					
	*p* value	0.964	0.688	0.574	0.411	**0.036**	–					
Caspase 3 mRNA	Spearman’s rho	0.293	−0.037	0.452	0.571	0.394	0.096	–				
	*p* value	***0.054***	0.808	**0.003**	**< .001**	**0.007**	0.566	–				
AIP mRNA	Spearman’s rho	0.442	0.291	0.378	0.391	0.200	−0.311	0.291	–			
	*p* value	**0.002**	**0.041**	**0.009**	**0.008**	0.187	***0.058***	***0.053***	–			
D2R mRNA	Spearman’s rho	0.477	0.415	0.354	0.318	0.225	−0.199	0.094	0.557	–		
	*p* value	**< .001**	**0.002**	**0.014**	**0.026**	0.137	0.231	0.539	**< .001**	–		
Ki67	Spearman’s rho	0.091	−0.110	−0.092	−0.382	0.295	0.512	−0.171	−0.205	−0.117	–	
	*p* value	0.526	0.438	0.541	**0.008**	***0.055***	**0.001**	0.273	0.162	0.408	–	
Age	Spearman’s rho	−0.339	0.082	−0.068	−0.012	−0.150	0.118	−0.286	−0.171	−0.068	0.001	–
	*p* value	**0.013**	0.555	0.647	0.934	0.324	0.480	***0.057***	0.234	0.625	0.994	–

Significant values are indicated in bold (nearly significant trends in *italics*)

Gene expression data obtained in the 2 groups are also summarized in Table 1. As shown in Fig. 1D–G, *SF1* mRNA expression did not significantly differ between the two groups and neither did *βLH*. In contrast, *βFSH* and *AIP* transcripts were significantly higher in group 1 as compared to group 2 (*P* = 0.042 and *P* = 0.024, respectively).

The expression of all genes was subsequently analyzed in a correlation matrix, including patients’ age and the Ki67 LI (Table 2). *SF1* was found to strongly and positively correlate with *βLH* and *D2R* expression (*r* = 0.506 and *r* = 0.477, *P* < 0.001 for both), to a lesser extent with *AIP* (*r* = 0.442, *P* = 0.002), but not with *βFSH* expression (*r* = 0.186, *P* = ns). No correlation was found between *SF1* and the Ki67 LI or any marker of the cell cycle (*CCND1, CCNA2, CCNB1*), except for a non-significative trend with *caspase* 3 (*r* = 0.293, *P* = 0.054), which also tended to decrease with age (*r* = −0.286, *P* = 0.057).

### Cell Cycle Markers and Tumor Characteristics

**Fig. 2:**
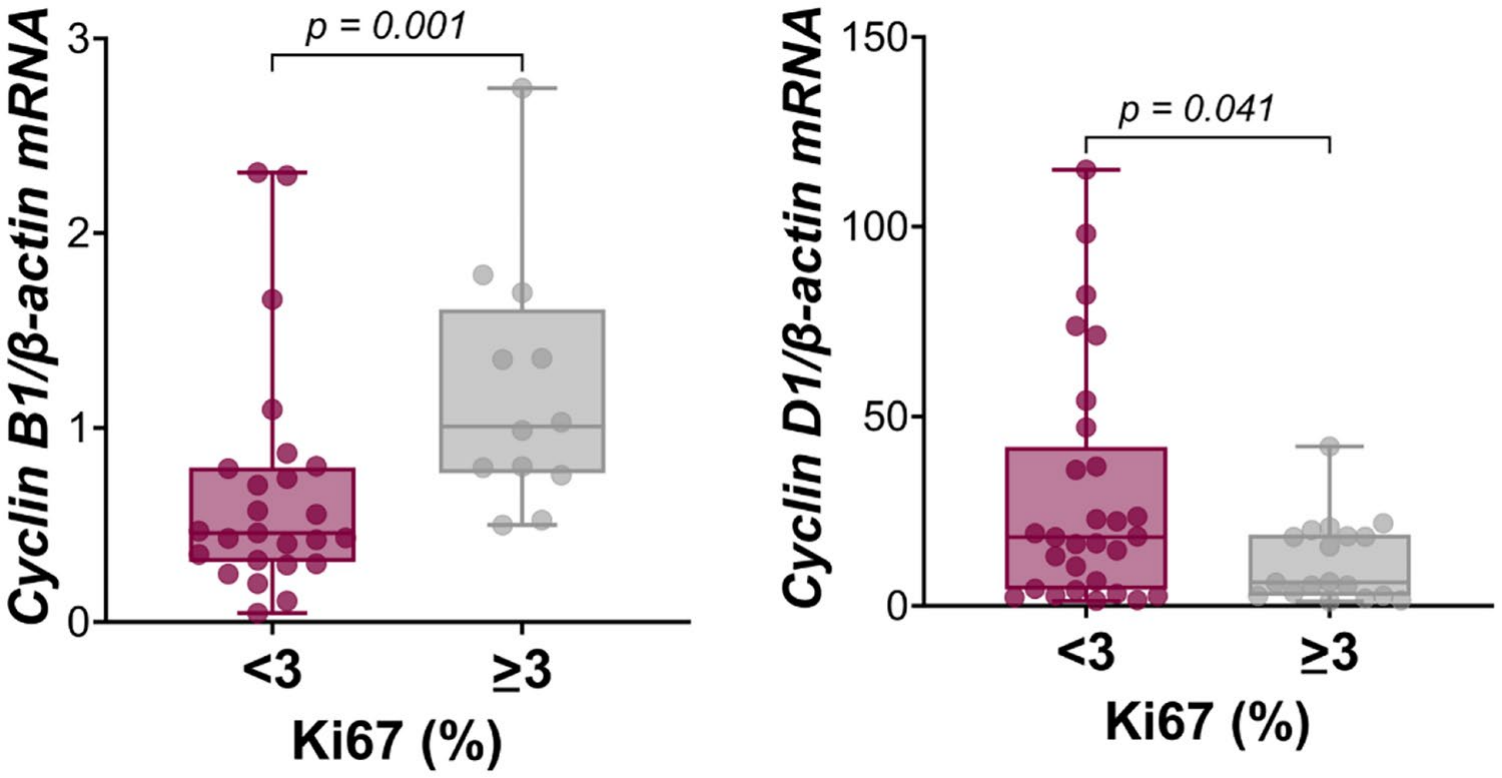
*CCNB1* and *CCND1* gene expression in GnPT according to the Ki67 LI. “High Ki67” GnPT had significantly higher *CCNB1* expression but significantly lower *CCND1* expression than “low Ki67” tumors

As compared with normal pituitaries, *CCNB1* was found to be upregulated (> 75° or 90° percentile) in 94.7% of cases, whereas *CCND1* and *CCNA2* were upregulated in 28.9% and 77.6%, respectively. The Ki67 LI was found to positively correlate with *CCNB1* (*r* = 0.512, *P* = 0.001) but negatively correlated with *CCND1* (*r* = −0.382, *P* = 0.008). Accordingly, as shown in Fig. 2, *CCNB1* expression was significantly higher but *CCND1* expression was significant lower in “high Ki67” than in “low Ki67” tumors (*P* = 0.002 and *P* = 0.041, respectively). In contrast, no significant correlation was found between the Ki67 LI and *CCNA2* or *caspase 3*, which did not differ between “high Ki67” and “low Ki67” tumors (data not shown). Similarly, the expression of gene encoding cyclins and the Ki67 LI were not significantly different in recurrent vs non-recurrent tumors, and recurrent GnPT were only characterized by a significantly lower *βLH* and *D2R* gene expression and a trend towards a lower *CCND1* expression (Supplementary Table 1).

### Factors Associated with AIP Expression

**Fig. 3:**
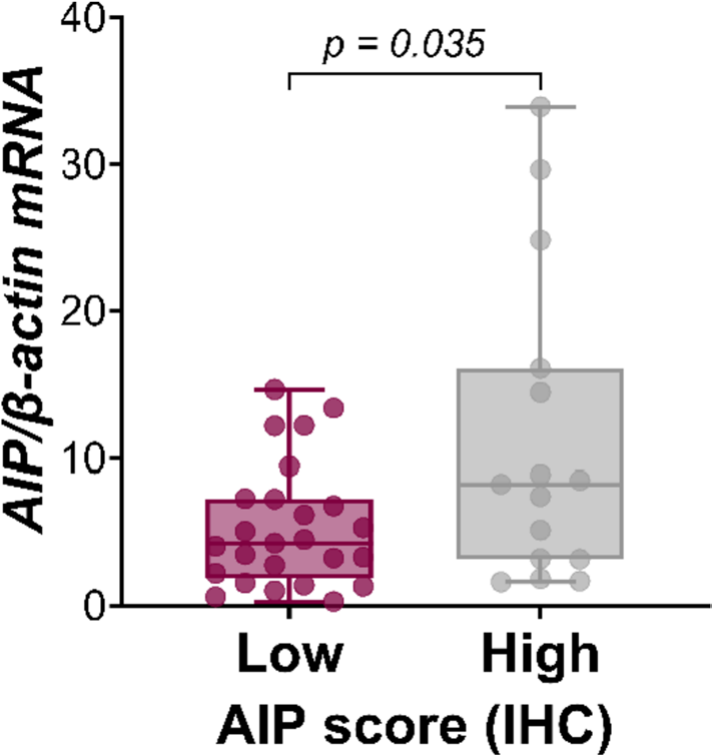
Correlation between AIP gene expression and immunostaining in GnPT. *AIP* transcripts were significantly higher in tumors expressing a high AIP immunostaining score (≥ 4)

Because, as reported above, *AIP* transcripts were found to be significantly lower in pSF1 tumors (*P* = 0.024 vs FSH/LH tumors), we further analyzed factors influencing AIP expression in GnPT. As shown in the correlation matrix, *AIP* transcripts were found to significantly and positively correlate with *βLH* (*r* = 0.391, *P* = 0.009), *βFSH* (*r* = 0.291, *P* = 0.041), and *CCND1* (*r* = 0.391, *P* = 0.008) but were unrelated to *CCNA2*, *CCNB1*, or the Ki67 LI (Table 2). Among the 40 cases studied for AIP-IHC, 29 (72.5%) showed AIP immunostaining, out of which 15 (37.5%) had a high AIP score. Although *AIP* transcripts were significantly higher in the presence of a high AIP score (*P* = 0.035 vs a low AIP score) (Fig. 3), the median AIP immunostaining score was similar in the 2 groups of GnPT (Table 1), and the proportion of tumors with a high AIP score was similar in “high Ki67” and in “low Ki67” tumors (33.3% vs 40%, respectively). This was confirmed in the FSH/LH subgroup (45.4% vs 40.9%, respectively).

In addition, no significant difference was found in the AIP gene or protein expression according to the patient’s gender, tumor size, invasiveness, or recurrence (data not shown).

Examples of AIP and SF1 immunostaining are shown in Supplemental Fig. 2.

### Factors Associated with D2R Expression

**Fig. 4:**
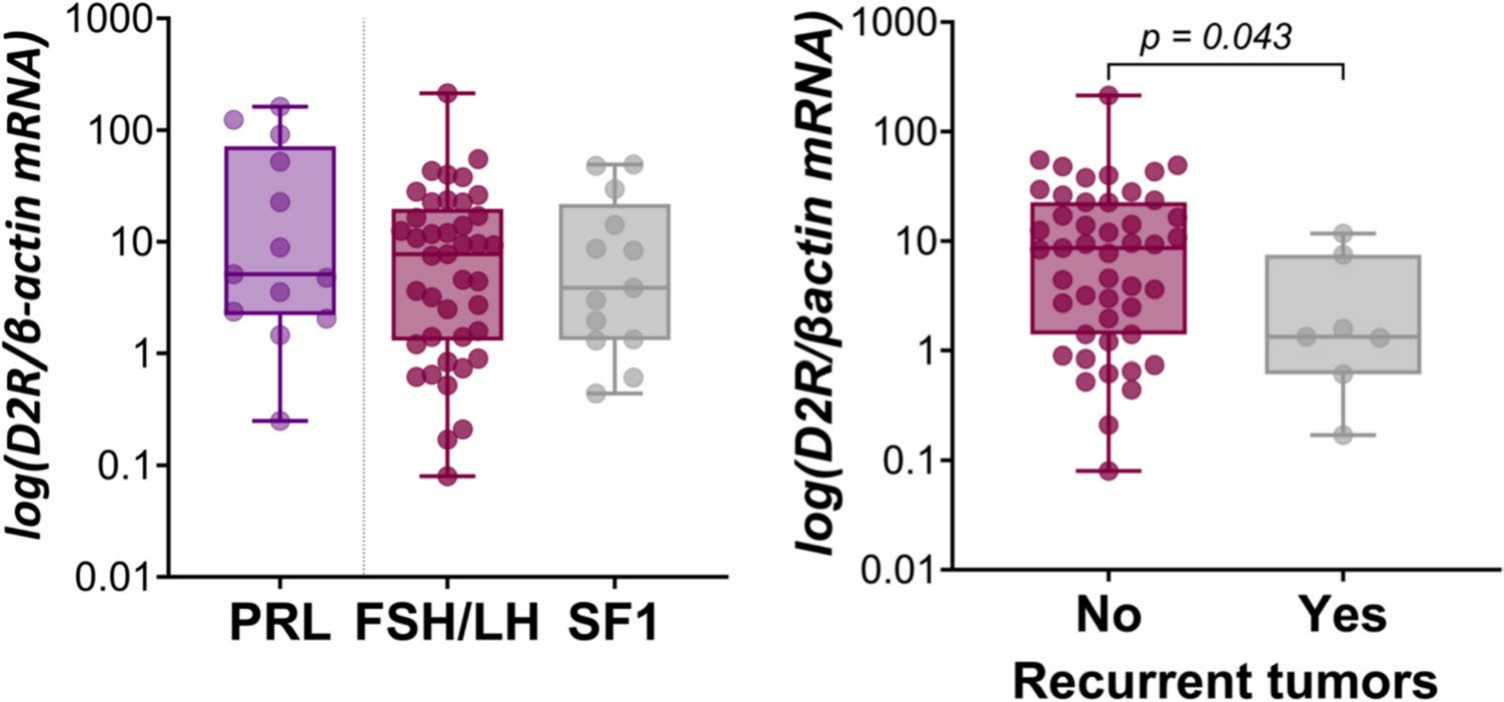
*D2R* gene expression in GnPT and functional lactotroph tumors. No significant difference in *D2R* expression was found between functional lactotroph tumors (PRL) and GnPT, or between FSH/LH and pSF1 phenotypes (**A**). In contrast, *D2R* transcripts were significantly lower in recurrent GnPT as compared to non-recurrent cases (**B**)

*D2R* transcripts were detectable in all cases, similar expression observed in a control series of functional lactotroph tumors (Fig. 4A), and significantly lower in recurrent cases (*P* = 0.043 vs non-recurrent cases) (Fig. 4B).

As shown in the correlation matrix, *D2R* transcripts were strongly and positively correlated with *SF1* and *AIP* (*P* < 0.001 for both), to a lesser extent with *βFSH* (*P* = 0.002), *βLH* (*P* = 0.014), and *CCND1* (*P* = 0.026) but not with *CCNB1*, *CCNA2*, *caspase 3*, or the Ki67 LI (Table 2). However, the difference between the 2 groups of GnPT did not reach significance, and no significant variations were found according to tumor size or invasiveness.

**Fig. 5:**
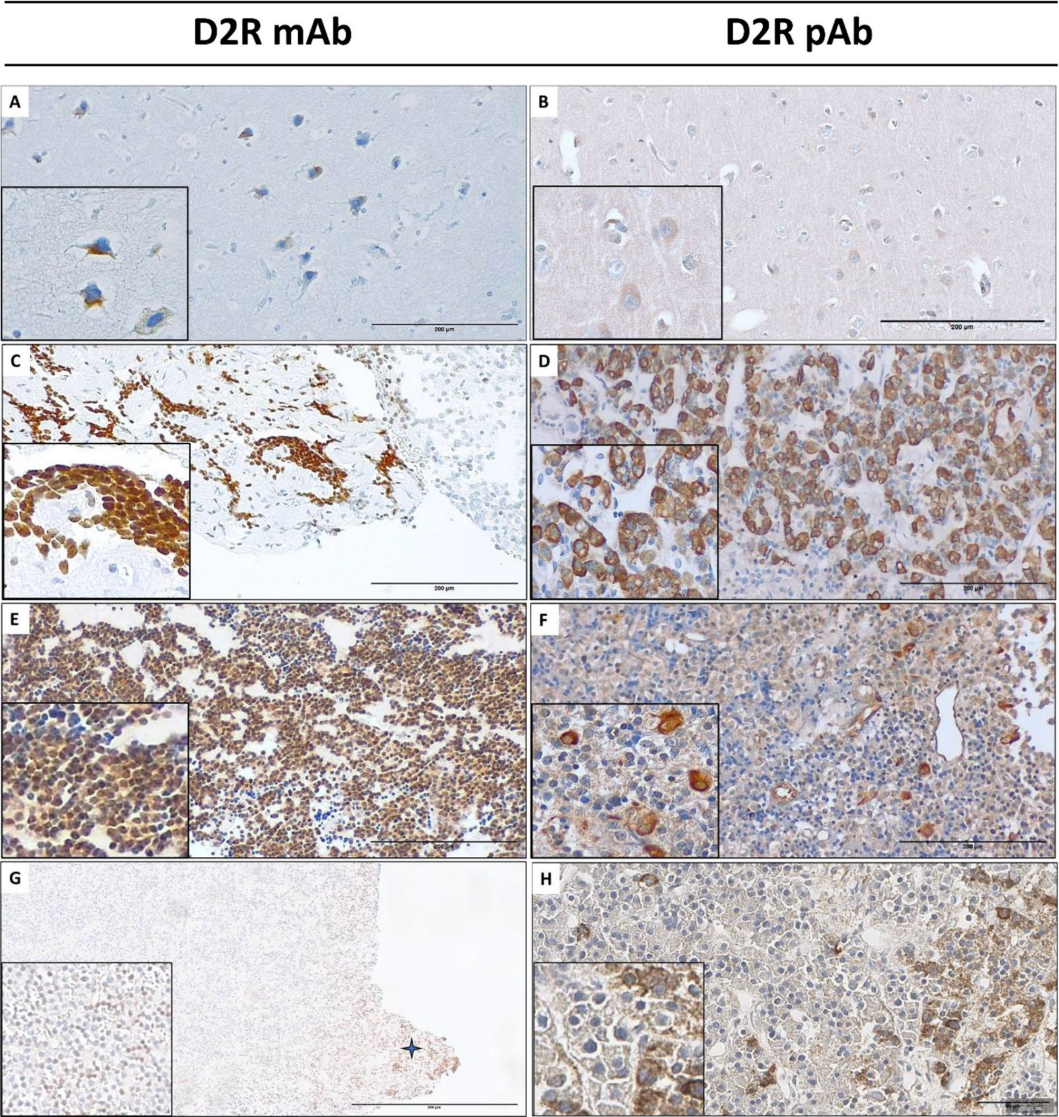
D2R immunostaining in controls and representative examples of GnPT using a monoclonal (D2R-mAb) or a polyclonal (D2R-pAb) antibody. In the brain tissue (**A**, **B**) (×200 magnification; inset at ×400), D2R staining was essentially cytoplasmic. In normal pituitary gland fragments, nuclear (**C**) or cytoplasmic with occasional membrane (**D**) D2R staining was observed (×200 magnification; inset at ×400). Similar results were obtained on functional lactotroph tumors (**E**, **F**) (×200 magnification; inset at ×400). Representative examples of D2R immunostaining in GnPT are also shown (**G**, **H**) using the mAb (**G**) nuclear staining which was observed in a normal juxta-tumoral pituitary fragment (indicated by a star) and in scattered neoplastic cells (inset) (×100 magnification; inset at ×400), whereas cytoplasmic staining was focally observed with the pAb (H) (×400 magnification)

Both the antibodies used for the evaluation of D2R protein expression by IHC—one monoclonal (D2R mAb) and one polyclonal (D2R pAb)—showed immunopositivity on a control brain sample (Fig. 5A, B). In such tissue, a clear cytoplasmic immunostaining was observed, with occasional membrane staining. However, in the study of normal pituitary fragments contaminating unselected samples of PitNETs, D2R immunostaining was mostly observed using the D2R pAb, which revealed cytoplasmatic but also membrane staining in a subset of cells (Fig. 5C, D). Unexpectedly, using the D2R mAb, D2R immunostaining on NP fragments was mostly nuclear. Individual data obtained with either antibody on a series of functional lactotroph tumors and GnPT are detailed in Supplemental Table 2, and representative examples are shown in Fig. 5E, F. Based on such data, no quantitative score could be proposed. To summarize, D2R pAb showed some degree of cytoplasmic staining in 8/9 functional lactotroph tumors, with a membrane staining in 5/9 cases, although immunopositivity was focal or scattered. On the contrary, a single case of functional lactotroph tumor showed diffuse cytoplasmic and nuclear immunostaining using the D2R mAb. In a subset of GnPT, representative of different levels of *D2R* gene expression, only some nuclear, and generally faint, immunopositivity was observed using the mAb. Instead, a clear immunopositivity was observed in 10/15 cases using the D2R pAb (Fig. 5G, H), although it appeared mostly cytoplasmic and detectable at membrane level in a single case.

## Discussion

To the best of our knowledge, this study is the first to provide a clinicopathological and molecular characterization of GnPT according to their immunoprofile. As compared to FSH/LH-immunopositive GnPT, pSF1 tumors tended to occur in older patients and displayed a lower gene expression of *βFSH* and *AIP* but did not significantly differ in terms of gender, tumor size, invasiveness, Ki67, or any marker of cell cycle or apoptosis. This is relevant since pSF1 tumors previously belonged to the previous “null cell” group of PitNETs, which are now defined by the absence of lineage-specific transcription factor expression [3, 4], so a re-evaluation of the potentially worse prognosis of “null cell” tumors among NFPT needs some re-evaluation in light of the pituitary lineage of origin of hormone-negative cases [2]. In a recent study, “null cell” pituitary tumors were still reported to be more at risk than GnPT (which included pSF1 cases), albeit due to the retrospective nature of the study, the expression of lineage-specific transcription factors was not available on all “null cell” tumors [22]. A lower, patchy expression of SF1 in GnPT was also found to be more frequently associated with post-operative recurrences than a higher, diffuse expression of SF1 and that the transcriptional profile of low SF1-expressing tumors suggested an increased activation of the PI3K/Akt pathway [23]. According to this study, the SF1 labeling index was more accurate than Ki67 in predicting short-term recurrences [23]. Nonetheless, data from the current study suggest that *SF1* gene expression is similar in the two groups of GnPT and that pSF1 tumors do not represent per se a distinct “high-risk” tumor phenotype. Of note, because Pit1 and Tpit expression were evaluated by RT-PCR in all samples, primarily to exclude contamination by normal pituitary fragments, we are sufficiently confident that only GnPT were included and that SF1 immunostaining was not the expression of a potential co-lineage [24]. Large follow-up studies would be useful to verify the recurrence risk of pSF1 tumors.

Another interesting finding of this study is that *SF1* gene expression was found to significantly decrease with patients’ age and significantly correlated with *βLH* but not with *βFSH* expression. The strong correlation between *SF1* and *βLH* (*P* < 0.001) is consistent with the direct transcriptional role of SF1 in the positive control of βLH expression reported in rodents, involving SF1-binding-sites in its 5′ flanking promoter region [25]. This has also been shown in the sheep pituitary, with an SF1-binding site being identified in the *β*LH but not in the *β*FSH promoter [26]. Nonetheless, SF1 may indirectly enhance *βFSH* transcription in normal gonadotrophs through GnRH stimulation [25, 27]. Receptors for sex steroids [28] and other transcription factors are also involved in the regulation of gonadotropins [29, 30]. The functional heterogeneity of GnPT was pointed out in a recent study which reported, based on quantitative immunohistochemistry for gonadotropins, a majority of cases showing both FSH and LH (74.5%) immunopositivity, followed by pure FSH- (21.4%) and a very minority of pure LH-immunopositive tumors (4.1%) [31]. Significant differences in the expression of ER*α* and the somatostatin receptor SST2 were found between these subgroups, suggesting that functional differences may reflect different pathogenetic mechanisms and have translational implications [31]. In the present study, no significant difference was found in *SF1* expression between FSH/LH and pSF1 tumors, and no correlation was found between *SF1* and the Ki67 LI or any marker of cell cycle, which may be in contrast with the recurrences observed in low SF1 expressing GnPT [23]. Because we did not perform SF1 immunostaining in the presence of unequivocal gonadotropin immunostaining, we could not assess this point, but the lower *βLH* transcripts observed in recurrent cases might be an indirect index of a lower SF1 transcriptional activity. On the other hand, we found that *SF1* gene expression was significantly correlated with that of *AIP* and *D2R*.

A strong positive correlation was indeed observed for the first time between *AIP* and the three markers of gonadotroph phenotype, *SF1*, *βLH*, and *βFSH*. The paradoxical AIP gene and/or protein expression was already reported in GnPT [17–19]. In the present study, AIP immunostaining was also observed in nearly 75% of the cases, nearly half of which with a high score. Although *AIP* transcripts were significantly higher in the presence of a high AIP score and in FSH/LH-GnPT, the AIP immunoscore was not significantly higher in this latter group as compared to pSF1 cases. Overexpression of mir-107 targeting *AIP* mRNA has been involved in NFPT and may account for some discrepancy between AIP gene and protein expression [32]. However, the mechanisms leading to paradoxical AIP expression in GnPT and its potential implications are unclear. No relationship has been found between AIP expression and tumor invasiveness [19, this study]. In contrast, high AIP immunostaining was previously reported in 83% of FSH/LH-GnPT with a high Ki67 (>3%) and 21% of those with a low Ki67, further suggesting an opposite pattern as compared to somatotropinomas [19]. We were unable to confirm such findings in a larger series of GnPT. Indeed, the proportion of tumors with a high AIP score was similar in “high Ki67” and in “low Ki67” tumors, and this was confirmed in the FSH/LH-immunopositive subgroup. *AIP* gene expression also appeared to be unrelated to the Ki67 LI. To further investigate the potential relationship between AIP and cell proliferation in GnPT, we also analyzed the relationship between the transcriptional expression of *AIP* and genes encoding cyclins found to be overexpressed in NFPT [33], namely cyclin D1 [34–39], cyclin A2 [40, 41], and cyclin B1 [42, 43]. A positive correlation was observed between the transcriptional levels of *AIP* and *CCDN1*, which encodes cyclin D1, a known target of extracellular stimulation by mitotic growth factors and consistently reported to be overexpressed in NFPT—as defined before the introduction of transcription factors to identify their lineage of origin [34–39]. Extra-cellular factors may thus contribute to increase AIP expression in GnPT. In contrast, we found AIP to be unrelated to *CCNA2* and negatively correlated with *CCNB1.* However, *CCNB1* was significantly higher in “high Ki67” tumors (≥ 3%), whereas *CCND1* was significantly higher in “low Ki67” cases (<3%), *CCNA2* being similar in both groups. Overall, these data argue against a role of AIP in the proliferative or invasive potential of GnPT. Therefore, if AIP is a well-documented tumor suppressor gene in somatotroph PitNETs, current data do not support a potential, opposite, oncogenic function for AIP in GnPT—as recently reported in some non-endocrine neoplasms [44]. This suggests that AIP expression has no prognostic value in GnPT, although follow-up studies may be useful to further validate this point.

This study also unraveled an intriguing discrepancy between *CCND1* and *CCNB1*, with opposite variations according to the Ki67 LI. *CCNB1* overexpression in GnPT has previously been associated with downregulation of miR-410, which decreases *CCNB1* transcription and enhances its degradation [45]. Because cyclin B1 is expressed in the G2/M transition and is essential for the initiation of mitosis [46], it is not surprising that *CCNB1* expression may be indicative of tumor proliferation. In contrast, cyclin D1 is involved early in the cell cycle, but initial progression into the cell cycle may not necessarily lead to proliferation. Our findings are reminiscent of the opposite role of cyclin B1/2 and cyclinD1 during neurogenesis, coordinating cortical progenitor self-renewal and lineage commitment [47]. In this model, cyclin D1 promoted cell differentiation [47]. An attractive hypothesis is that, in GnPT, cyclin D1 and cyclin B1 may also contribute to differentially control cell function and proliferation, respectively. Further in vitro studies would be useful to explore such hypothesis and clarify the potential prognostic role of cyclin D1 overexpression, which is a preferential feature of NFPT [34–39]. Indeed, correlations between cyclin D1 immunostaining and the Ki67 LI and tumor volume and cavernous sinus invasion were observed on a representative series of PT including NFPT but not specifically detailed in this subgroup [39], and the same occurred in a subgroup of aggressive PT [36]. The highest cyclin D LI reported in NFPT compared to other phenotypes was observed for both recurrent and tumors [33]. In addition, in our series, *CCND1* expression tended to be lower in recurrent cases, but cyclin D1 has a short half-life, and protein overexpression appears to be generally modest [34–36], and not correlated with mRNA levels [34]. Therefore, the impact of our findings on cyclin D1 protein expression should be further evaluated. *CCNA2*, which encodes cyclin A2, involved at an intermediate stage of the cell cycle, was modestly correlated with *CCNB1* and tended to correlate with the Ki67 LI but was unrelated to *CCND1, AIP*, or any marker of gonadotroph phenotype. The cyclin B/A immunostaining ratio was also previously reported to be higher in nonfunctioning pituitary tumors that regrew compared to those who did not [33]. Overall, it appears that cyclin B1/*CCNB1* may be the best marker of cell proliferation in GnPT, and further studies, including immunohistochemistry and prospective follow-up, could be designed to evaluate its potential prognostic value in such tumors.

The expression of D2R was also investigated because of its potential translational impact. We found *D2R* gene expression to be significantly correlated with that of *AIP,* the three markers of gonadotroph phenotype—in particular *SF1—*and to a lesser extent *CCND1*. In a recent preliminary report, *D2R* mRNA appeared to be lower in pSF1 than in gonadotropin-expressing GnPT [48], but this point was not confirmed when enlarging the series for the final study, and median D2R levels were also similar among the 2 groups of GnPT and in functional lactotroph tumors taken as controls. However, D2R expression was found to be significantly lower in recurrent GnPT. In one study comparing the efficacy of post-operative dopamine-agonist drugs given either as a preventive treatment of tumor regrowth or as a remedial treatment in recurrent cases, additional surgery or radiotherapy was required in 13% of the preventive group, versus 38% of the remedial group and 42% of the untreated control group [13]. Taken together, these data suggest that the potential response to DA may decrease during tumor progression. The present study points out that a major limitation in the study of D2R protein expression in PitNETs is the poor reliability of current IHC procedures, with limited evidence of membrane staining. Another limitation was the heterogeneity of tumor staining, which was found also on functional lactotroph tumors. Thus, despite the use of two antibodies in different experimental conditions, we were unable to set a quantitative score for D2R immunostaining and reliably analyze the protein correlates of *D2R* gene expression. An interesting finding, however, is that membrane staining, which could be observed on control sections (brain, normal pituitary, functional lactotroph tumors) was exceptionally found in GnPT, and only using the polyclonal antibody. If *D2R* transcripts were previously reported to be higher in DA-responsive NFPT, especially in their short isoform [11], the predictive value of D2R immunostaining is controversial and overall disappointing. Some informative value was suggested in one study [12] but not by others [13, 49], D2R staining being mostly cytoplasmic in all studies. Defective anchorage of D2R (as well as SSTRs) in PitNETs may be due to low or absent filamin-A expression and account for resistance to DA, although this has not been specifically studied in GnPT [50]. Therefore, if focal expression and inappropriate D2R localization may contribute to the inconstant and moderate response of GnPT to DA in clinical practice, the current predictive value of D2R immunostaining is poor and discourages its use for the selection of potentially responsive patients, and technical optimization is warranted.

In conclusion, this study suggests that pSF1 tumors tend to develop later in life as compared to gonadotropin-expressing GnPT, but do significantly differ from this latter group—which is the most commonly encountered—in terms of macroscopic characteristics, Ki67, and *SF1* or cyclin gene expression. AIP expression in GnPT appeared to be unrelated to invasion or proliferation. D2R may be mislocalized in GnPT, but D2R immunostaining still requires optimization. Thus, at the moment, AIP and D2R immunostaining appear to be poorly relevant for clinical practice in these tumors. In contrast, cyclin B1 appears as a promising marker of proliferation in GnPT, and its potential prognostic value would deserve further studies. Similarly, the potential prognostic value of cyclin D1 immunostaining in NFPT should be further clarified.

### Supplementary Information

ESM 1 (DOCX 1854 kb)

## Data Availability

Raw data are available at the Department of Biotechnological and Applied Clinical Sciences, University of L’Aquila, and at the Neuromed IRCCS, Pozzilli, Italy.
