# Supplementary material for: Clinical and Molecular Characteristics of Gonadotroph Pituitary Tumors According to the WHO Classification
Source: Endocr Pathol. 2023 Dec 14;35(1):1–13. doi: 10.1007/s12022-023-09794-w (PMC10944444; doi:10.1007/s12022-023-09794-w)
Supplement: Supplementary file 1 — (DOCX 1854 kb) [file 12022_2023_9794_MOESM1_ESM.docx]

Supplementary Materials

|  | **Recurrent**  **(n=7)** | **Non-recurrent**  **(n=47)** | **p** |
| --- | --- | --- | --- |
| Gender | 5 M; 2 F | 32 M; 15 F | ns |
| Age (yrs) | 67 [58-83] | 57 [36-76] | *0.071* |
| Tumor size [cm] | 2.85 [1.40-4.20] | 3.00 [1.60-5.30] | ns |
| Invasive tumors | 3/7 (42.9%) | 29/45 (64.4%) | ns |
| Ki67 (%) | 3.00 [1.00-5.00] | 2.00 [0.100-8.00] | ns |
| *SF1 mRNA* | 12.2 [2.24-492] | 21.0 [0.127-294] | ns |
| *βLH mRNA* | 11.6 [0.02-53.9] | 33.9 [0.232-4820] | **0.042** |
| *βFSH mRNA* | 19.7 [1.89-152] | 52.8 [0.426-1067] | ns |
| *CCND1 mRNA* | 2.63 [1.94-20.8] | 16.5 [1.18-115] | *0.091* |
| *CCNA2 mRNA* | 0.0537 [0.0271-0.73] | 0.0830 [0.01-1.81] | ns |
| *CCNB1 mRNA* | 1.03 [0.248-1.78] | 0.555 [0.0460-2.75] | ns |
| *Caspase 3 mRNA* | 0.08 [0.01-0.45] | 0.30 [0.01-1.98] | ns |
| *D2R mRNA* | 1.33 [0.174-11.7] | 8.62 [0.0849-213] | **0.043** |
| *AIP mRNA* | 3.87 [1.53-10.9] | 5.33 [0.290-33.9] | ns |
| AIP score (IHC) | 3.50 [3-4] | 3.00 [1-6] | ns |

**Supplemental Tab.1:** Bio-clinical and molecular characteristics of recurrent GnPT as compared with non-recurrent tumors.

*Legend*: IHC: immunohistochemistry

| **N** | **Sex** | **Age** | **PitNET** | **Pre-op DA treatment** | **D2R mAb** | **D2R pAb** |
| --- | --- | --- | --- | --- | --- | --- |
| 1 | M | 52 | PRL (Ma) | No | Negative | Scattered (c, mb) |
| 2 | M | 32 | PRL (Ma) | No | Negative | Focal (c) |
| 3 | M | 28 | PRL (Ma) | Yes | Negative | Focal (c) |
| 4 | F | 34 | PRL (m) | Yes (R) | Negative | Scattered (c,mb) |
| 5 | F | 34 | PRL (m) | Yes (I) | Negative | Focal (c,mb) |
| 6 | M | 26 | PRL (Ma) | No | Negative | Scattered (c) |
| 7 | F | 21 | PRL (m) | Yes (R) | Negative | Scattered (c,mb) |
| 8 | M | 19 | PRL (Ma) | Yes (R) | Diffuse (c,n) | Scattered (c,mb) |
| 9 | F | 34 | PRL (Ma) | No | Negative | Negative |
| 10 | F | 70 | FSH/LH | No | Diffuse (c) | *NA* |
| 11 | F | 47 | FSH/LH | No | Negative | Diffuse (c) |
| 12 | M | 40 | FSH/LH | No | Negative | Focal (c,mb) |
| 13 | M | 41 | FSH/LH | Yes | Negative | *NA* |
| 14 | F | 46 | FSH/LH | No | Negative | *NA* |
| 15 | M | 70 | FSH/LH | No | Negative^1^ | *NA* |
| 16 | F | 41 | FSH/LH | No | Negative | Negative |
| 17 | F | 45 | FSH/LH | Yes | Negative | Scattered (c) |
| 18 | M | 46 | FSH/LH | No | Negative^1^ | Focal (c) |
| 19 | M | 62 | FSH/LH | NA | Negative | Negative |
| 20 | M | 58 | FSH/LH | Yes | Negative^1^ | Focal (c) |
| 21 | M | 66 | FSH/LH | No | *NA* | Diffuse (c) |
| 22 | M | 66 | FSH/LH | No | *NA* | Negative |
| 23 | F | 52 | FSH/LH | No | *NA* | Focal (c) |
| 24 | M | 52 | FSH/LH | No | *NA* | Negative |
| 25 | F | 58 | FSH/LH | No | *NA* | Focal (c) |
| 26 | M | 72 | FSH/LH | No | *NA* | Negative |
| 27 | M | 49 | SF1 | No | Negative^1^ | Negative |
| 28 | F | 73 | SF1 | No | Negative^1^ | *NA* |
| 29 | M | 73 | SF1 | No | Negative^1^ | Focal (c) |
| 30 | M | 60 | SF1 | NA | Scattered (n) | *NA* |
| 31 | M | 56 | SF1 | Yes | *NA* | Focal (c) |

**Supplemental Tab. 2** D2R immunostaining in representative cases of PitNETs

*Legend:* D2R immunopositivity and localization in individual cases of GnPT and micro-(m) / macro- (M) functional lactotroph tumors (PRL) taken as controls. (I): intolerant to dopamine-agonist treatment; (R): resistant to dopamine-agonist treatment. (c): cytoplasmic , (n):nuclear and/or (mb) membrane staining : (^1^) faint nuclear positivity


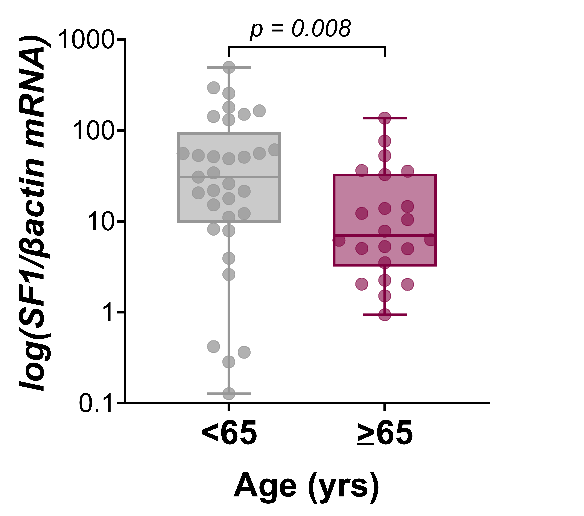


**Supplemental Fig.1** SF1 gene expression according to patients age.

*Legend:* *SF1* expression was significantly higher in younger patients (< 65 *vs* > 65 yrs-old)

*
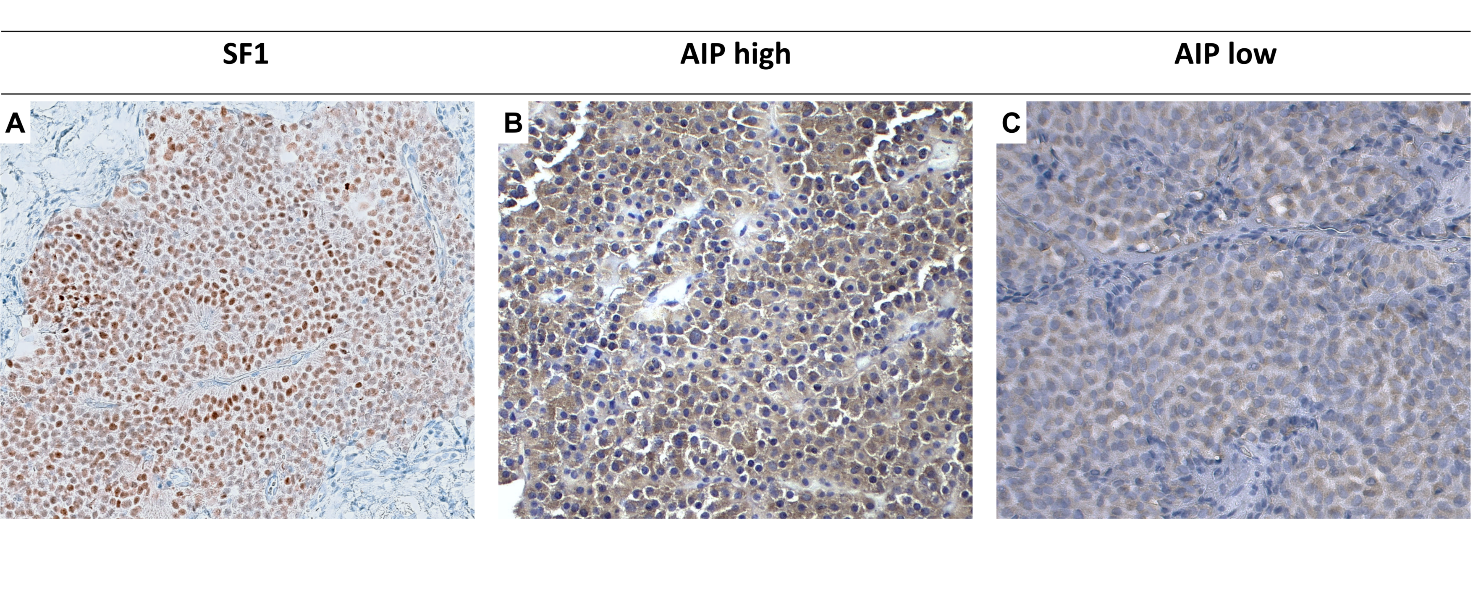
***Supplemental Fig. 2** Representative examples of SF1 and AIP expression in GnPT

*Legend:* Shown are examples of SF1 (A) and AIP high (B) or AIP low (C) immunostaining (200x). Immunopositivity for SF1 was exclusively nuclear (A) whereas AIP was purely cytoplasmic, regardless of AIP score (high score > 4 as defined in the text)
